# Sitosterolemia due to compound heterozygous mutations in *ABCG5*: a case report

**DOI:** 10.1186/s13256-025-05814-x

**Published:** 2026-01-28

**Authors:** Li-Li Wu, Li Zhang, Ting-Ting Tang, Yuan-Yuan Zhou, Xin-Xia Chang, Wei Guo, Hong-Mei Yan, Huan-Dong Lin

**Affiliations:** 1 Department of Endocrinology and Metabolism, Zhongshan Hospital, Fudan University, No.180 Fenglin Road, Shanghai, 200032 People’s Republic of China; 2 Department of Endocrinology and Metabolism Nephrology, Guangxi Medical University Cancer Hospital, Nanning, People’s Republic of China; 3 Department of Laboratory Medicine, Zhongshan Hospital, Fudan University, No.180 Fenglin Road, Shanghai, 200032 People’s Republic of China; 4 Department of Endocrinology, Yancheng No. 1, People’s Hospital, Yancheng, People’s Republic of China; 5 Department of Endocrinology and Metabolism, Sixth Affiliated Hospital of Kunming Medical University, Yuxi, People’s Republic of China

**Keywords:** Sitosterolemia, *ABCG5*, Cholesterol, Xenosterols, Case report

## Abstract

**Background:**

Sitosterolemia is an autosomal recessive genetic disorder characterized by hypercholesterolemia and tendon/hip xanthomas, primarily caused by pathogenic mutations in the *ABCG5* or *ABCG8* gene.

**Case Presentation:**

We report the clinical features and therapeutic outcomes of a 29-year-old Chinese male patient diagnosed with sitosterolemia. The patient first presented with xanthomas in childhood and subsequently developed recurrent hypercholesterolemia in adulthood. Genetic sequencing identified two distinct compound heterozygous mutations in the *ABCG5* gene: c.1256G>A (p.Arg419His) and c.751C>T (p.Gln251*). Lipoprotein profiling, including targeted analysis of plant sterols (xenosterols), was performed using gas chromatography–mass spectrometry. Following poor response to statin therapy, the patient was initiated on a low-plant-sterol and low-cholesterol diet, combined with ezetimibe (an NPC1L1 inhibitor). Serum total cholesterol and low-density lipoprotein cholesterol levels normalized within 2 months of this intervention.

**Conclusion:**

This case expands the mutational spectrum of sitosterolemia and provides valuable insights for the diagnosis and clinical management of this rare disorder.

## Introduction

Sitosterolemia (STSL) was first described by Bhattacharyya and Connor in 1974 [1], As an uncommon autosomal recessive disorder of lipid metabolism [2], it is characterized by marked elevations in serum plant sterol levels, the formation of tendon and tuberous xanthomas, and a predisposition to premature coronary atherosclerosis. Pathogenic mutations in the autosomal adenosine triphosphate -binding cassette subfamily G member 5 (*ABCG5*) or adenosine triphosphate-binding cassette subfamily G member 8 (*ABCG8*) genes are well established as the etiological basis of STSL [3]. The global prevalence of STSL is estimated to be 1 in 1,000,000–5,000,000 individuals, with approximately 200 cases reported worldwide to dated [4].

Sterols, including ergosterol, phytosterols, and cholesterol, are important naturally occurring bioactive compounds found in various organisms [5]. Phytosterols are the primary components of plant cell membranes and are abundant in nuts, grains, fruits, vegetables, vegetable oils, and cereals—with cereal sterols being the most prevalent [6]. Under physiological conditions, most phytosterols (particularly xenosterols) are excreted into the bile and intestinal lumen via the action of the *ABCG5*/*ABCG8* transporter complex, resulting in minimal intestinal absorption (<5%). Pathogenic mutations in *ABCG5* or *ABCG8* impair the normal excretion of phytosterols, leading to their systemic accumulation.

Herein, we report a rare case of sitosterolemia in a 29-year-old Chinese male with a history of xanthomas. The patient presented with hypercholesterolemia that was refractory to statin therapy and harbored two distinct variants in the *ABCG5* gene: c.1256G>A (p.Arg419His) and c.751C>T (p.Gln251*). This report focuses on the diagnosis, clinical manifestations, and management of sitosterolemia, while also expanding the spectrum of known *ABCG5* variants associated with this disorder.

## Case presentation

A 29-year-old Chinese male was referred to the Department of Endocrinology, Zhongshan Hospital, Fudan University in December 2021 for evaluation of persistent hypercholesterolemia. His parents were not consanguineous, and the patient was born to a healthy family. There were no significant perinatal adverse events, and he had no remarkable personal medical history. The family history was unremarkable for dyslipidemia, xanthomas, early-onset cardiovascular disease, or sudden unexplained death. At 9 years of age, the patient’s parents first noticed xanthomas on the dorsa of his hands and knees. These lesions were treated with laser cosmetic surgery, and no lipid profile testing was performed at that time. No recurrence of xanthomas has been observed to date. The patient maintains a regular exercise routine and reports no other health concerns. He denies a history of chest pain or exercise intolerance, and has never undergone evaluations for cardiovascular disease.

**Fig. 1:**
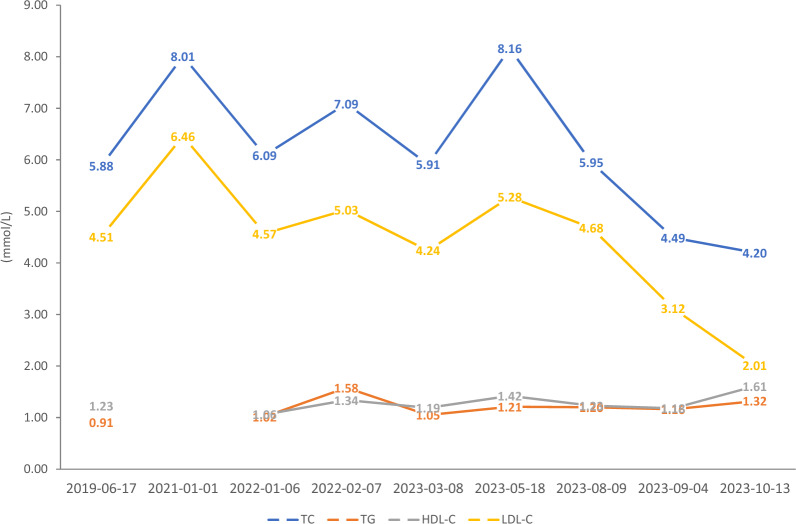
Serial biochemical and lipid laboratory values of the proband over time

**Fig. 2:**
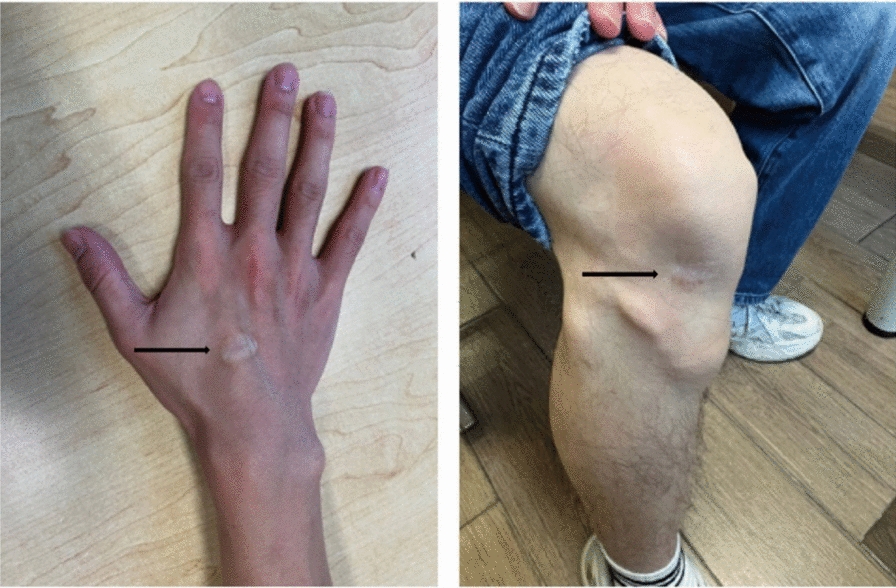
Old scars on the dorsum of the right hand and knee joint

In 2019, routine physical examination and blood tests revealed elevated cholesterol levels in the patient (Fig. 1). Physical examination findings were as follows: height 165 cm, weight 51 kg, body mass index (BMI) 18.73 kg/m^2^; old scars on the dorsum of the right hand and knee joints (Fig. 2); no new xanthomas, subcutaneous petechiae, or ecchymoses were observed across the body. Cardiac examination was unremarkable, with no signs of premature cardiovascular disease. Lungs, liver, spleen, and lymph nodes showed no abnormalities. Additionally, there were no underlying medical conditions (e.g., hypothyroidism, nephrotic syndrome) that could account for secondary hypercholesterolemia.

The patient was subsequently diagnosed with mixed hypercholesterolemia and initiated on rosuvastatin 10 mg once daily. However, 13 months of treatment yielded minimal changes in serum total cholesterol (T-Chol) and low-density lipoprotein cholesterol (LDL-C) levels. He was then switched to atorvastatin 5 mg/day combined with a high-fiber, low-fat, and low-cholesterol diet, but LDL-C and T-Chol remained persistently elevated. Imaging investigations showed: (1) 13 December 2021: carotid color Doppler ultrasound revealed smooth blood flow in both carotid arteries with no plaque formation; (2) 17 June 2023: follow-up carotid color Doppler ultrasound demonstrated plaque formation at the bifurcation of the right carotid artery and mild intimal thickening of the bilateral carotid arteries.

**Fig. 3:**
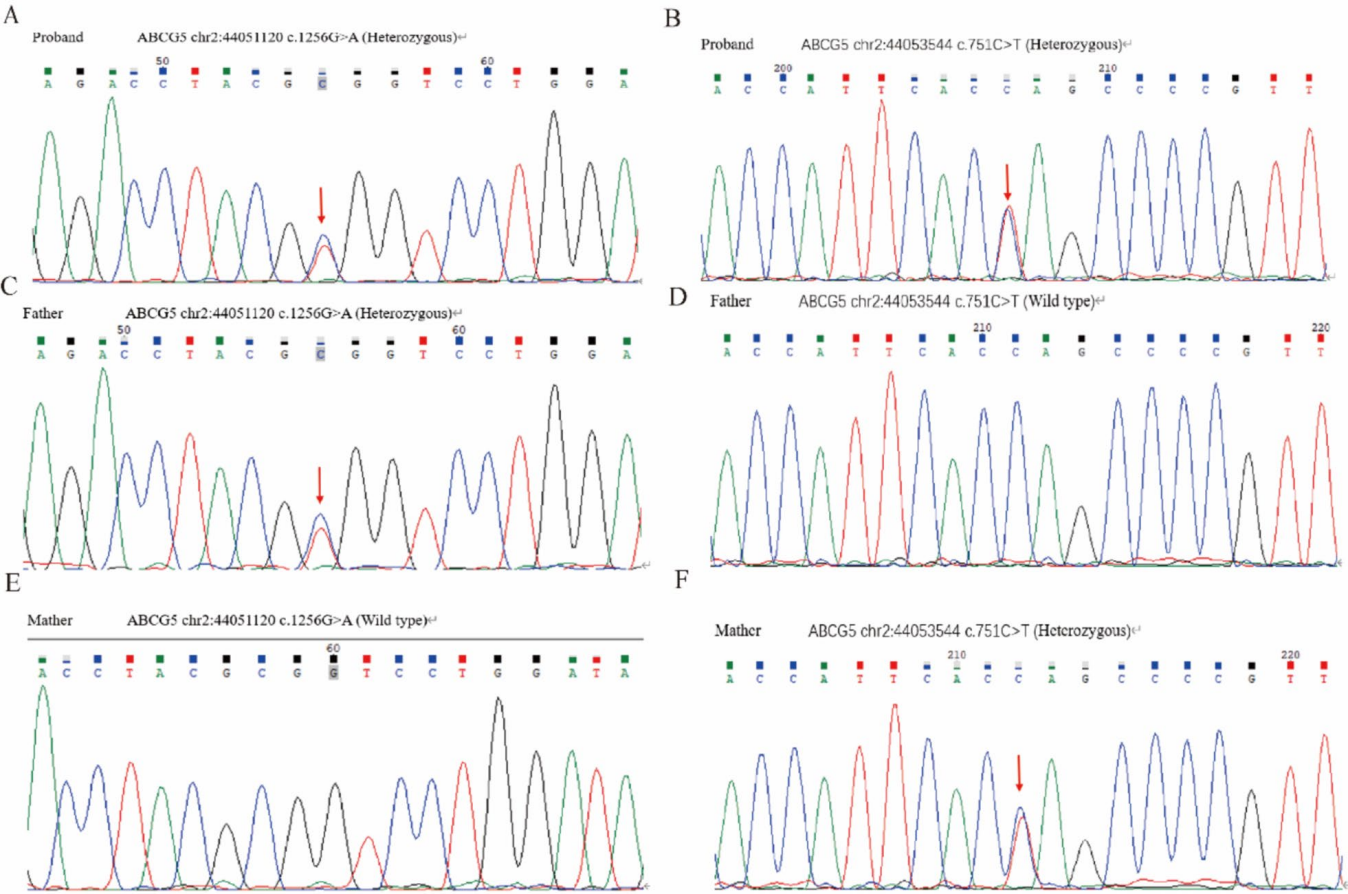
DNA sequence analysis of the *ABCG5* gene in the proband and his parents. (1) The proband harbored compound heterozygous variants in ABCG5: a heterozygous c.1256G>A (p.Arg419His) variant in exon 9, and a heterozygous c.751C>T (p.Gln251*) variant in exon 6; (2) The father carried a heterozygous c.1256G>A variant (ABCG5 chr2:44051120) in exon 9; (3) The mother carried a heterozygous c.751C>T variant (ABCG5 chr2:44053544) in exon 6

**Fig. 4:**
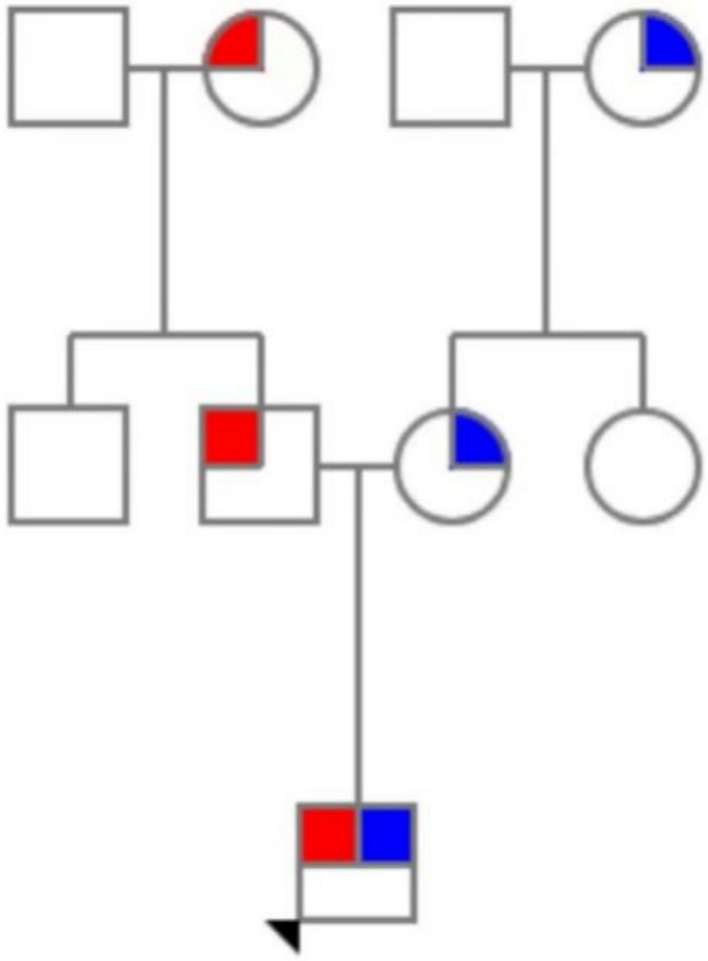
Pedigree of the family with sitosterolemia. Red symbols indicate carriers of the ABCG5 c.1256G>A variant (chr2:44051120); blue symbols indicate carriers of the ABCG5 c.751C>T variant (chr2:44053544). The arrow denotes the proband; squares represent males, and circles represent females

Given the patient’s persistently elevated cholesterol levels disproportionate to his BMI, childhood history of xanthomas, and poor response to statin therapy, genetic testing was performed to further guide clinical management. Whole-exome sequencing (WES) was conducted at the Department of Genetics, Zhongshan Hospital, Fudan University (Shanghai, China). Genetic analysis identified two compound heterozygous mutations in the *ABCG5* gene: a missense mutation c.1256G>A (p.Arg419His) and a nonsense mutation c.751C>T (p.Gln251*). Segregation analysis confirmed that each mutation was inherited in a heterozygous state from one parent (Fig. 3). No pathogenic mutations were detected in the *ABCG8* gene. Subsequent cascade screening of the patient's family revealed five heterozygous carriers of the identified *ABCG5* mutations, with no other family members manifesting clinical symptoms of sitosterolemia (Fig. 4).

Upon diagnosis of sitosterolemia, the patient was initiated on combination therapy consisting of ezetimibe 10 mg once daily and a strict low-cholesterol, low-plant-sterol diet. Approximately 4 weeks following the start of this intervention, marked reductions in his serum T-Chol and LDL-C levels were observed (Fig. 1).

**Fig. 5:**
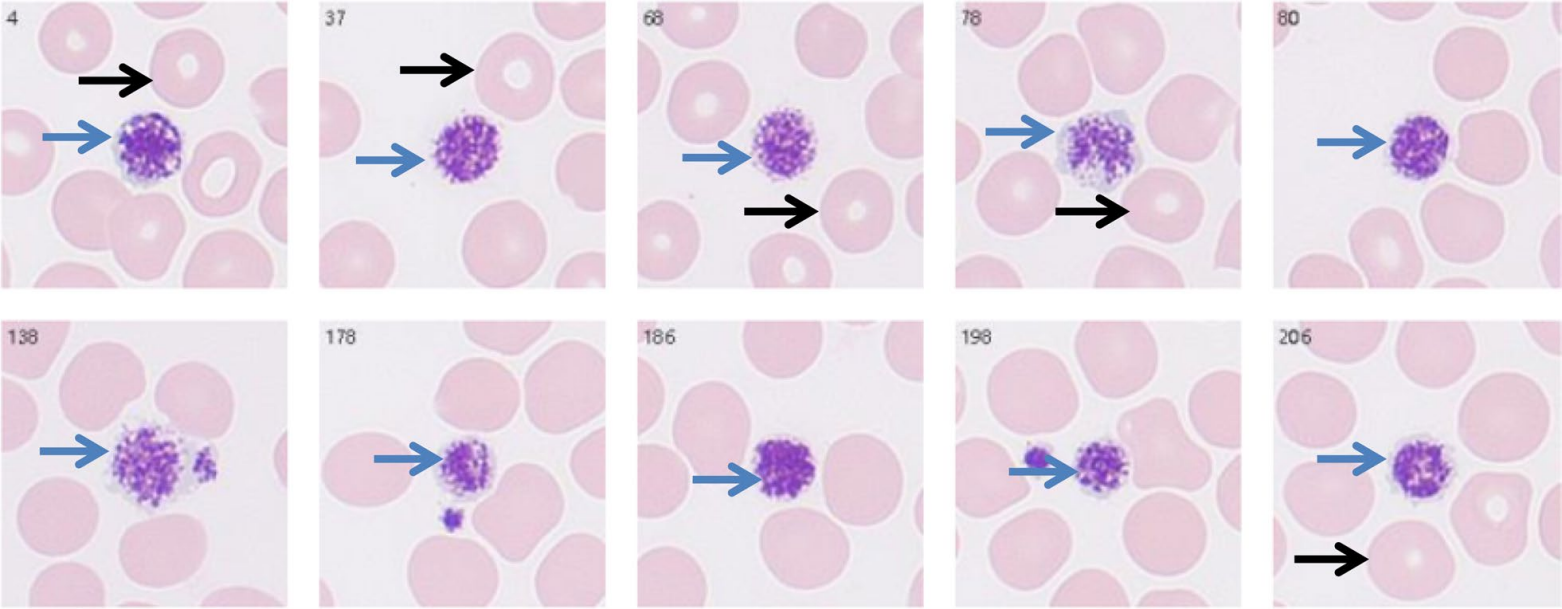
Peripheral blood smear. Blue arrows indicate large platelets (larger than erythrocytes), and black arrows indicate stomatocytes

With the patient’s informed consent, plasma plant sterol analysis was performed using high-performance liquid chromatography-diode array detection (HPLC–DAD) at the Shanghai Institute of Pediatric Research (Shanghai, China) during the follow-up period (Table 1). This assay is critical for confirming the diagnosis of sitosterolemia and evaluating therapeutic efficacy, as plant sterol accumulation is a hallmark of the disease. Additionally, macrothrombocytes were detected on follow-up laboratory examinations (Fig. 5).

**Table 1:** Quantitative analysis of serum plant sterols by HPLC–DAD

Plant sterols (2023–10-29)	Squalene	Dihydrocholesterol	Dehydrocholesterol	7-Dehydrocholesterol	Campesterol	Stigmasterol	β-Sitosterol
(μmol/L)	0.84	**32.46**	3.64	2.93	**134.05**	**14.14**	**246.08**
Reference value	0.23–1.54	4.44–12.53	2.81–8	1.71–8.51	5.39–20.81	0.09–1.08	3.74–14.58

To investigate whether heterozygous carriers of the patient’s identified *ABCG5* mutations exhibit alterations in blood cholesterol levels, fasting serum lipid profiles were measured in both parents (Table 2). Both parents had slightly elevated serum T-Chol and LDL-C levels compared with the normal reference range. Given their middle-aged status, age-related hyperlipidemia could not be excluded. They were therefore initiated on statin therapy, and follow-up testing 1 month later showed normalization of their serum lipid levels.

**Table 2:** Comparison of serum lipid concentrations among the proband and his parents

	TC (mmol/L) (<5.2)	TG (mmol/L) (<1.7)	HDL-C (mmol/L) (>1.04)	LDL-C (mmol/L) (<3.4)
Proband, 18 May 2023	8.16	1.21	1.42	5.28
Paternal, 8 April 2023	6.55	2.45	0.84	4.19
Maternal, 8 April 2023	6.28	1.19	1.61	3.51

## Discussion

Herein, we report a case of sitosterolemia in a patient who presented with a history of xanthomas during childhood and extreme hypercholesterolemia in adulthood. Accurate diagnosis of sitosterolemia is crucial for formulating appropriate treatment plans, determining precise prognoses, initiating early interventions, and preventing potentially life-threatening complications in the long term.

Hypercholesterolemia is a common clinical condition. However, when there is a discrepancy between the severity of hypercholesterolemia and the patient’s age and BMI, or when statin therapy proves ineffective, alternative etiologies should be considered. In this case, secondary hypercholesterolemia was first excluded, and the patient was routinely initiated on statin therapy, which yielded no significant therapeutic effect. Subsequent detailed medical history-taking revealed that the patient had no history of unusual dietary habits but had developed xanthomas at the age of 9 years. Combined with the patient’s BMI and physical characteristics, these clinical findings prompted consideration of specific underlying causes (e.g., sitosterolemia).

The main clinical manifestations of sitosterolemia include liver dysfunction, xanthomas, hypercholesterolemia, and accelerated atherosclerosis [7]. Hematological abnormalities have also been reported, such as stomatocytosis, hemolysis, anemia, and macrothrombocytopenia [8]. Clinically, sitosterolemia exhibits substantial phenotypic variability, ranging from asymptomatic presentations to early cardiac death [9]. Most patients with sitosterolemia are diagnosed during childhood owing to the presence of xanthomas; in contrast, some cases are misdiagnosed as familial hypercholesterolemia, leading to inappropriate treatment [10]. Sitosterolemia has been documented as early as 3 months of age, with pediatric cases reported more frequently than adult ones [11]. Additionally, some patients with mild symptoms may not present with hypercholesterolemia until adulthood. Owing to the lack of awareness regarding the potential of sitosterolemia, these adult patients are often not diagnosed correctly.

Accurate diagnosis of sitosterolemia is hindered by two key factors: the variable clinical manifestations of the disease and the inability of routine biochemical assays to distinguish between phytosterols and cholesterol [12]. Gas chromatography–mass spectrometry (GC–MS) is currently recognized as the gold-standard diagnostic method for detecting phytosterols in blood; however, this technique has not been widely adopted for phytosterol testing in many hospitals and regions. Specifically in China, clinical laboratories have not implemented GC–MS for plant sterol measurement, and no standardized reference intervals for plant sterols tailored to the Chinese population have been established to date [12].

Subsequently, genetic testing was performed to further guide the patient’s clinical management. Ultimately, the patient was found to carry heterozygous mutations in the *ABCG5* gene. Specifically, two heterozygous mutations were identified in the patient’s *ABCG5* gene: a heterozygous c.1256G>A (p.Arg419His) mutation located on exon 9, which was inherited from the father; and a heterozygous c.751C > T (p.Gln251*) variant on exon 6, which was inherited from the mother. A literature review revealed that cases involving each of these two mutations—either occurring alone or in combination with other mutations—have been previously reported [12, 13]. However, simultaneous occurrence of these two specific heterozygous mutations in the *ABCG5* gene has not been previously documented as a potential etiological factor for sitosterolemia.

Regarding gene mutations associated with sitosterolemia, mutations in the *ABCG8* gene are more prevalent in other populations, whereas mutations in the *ABCG5* gene are more common among patients of Asian descent—particularly in Chinese and Japanese individuals [11, 13]. Clinically, sitosterolemia exhibits marked heterogeneity; however, xanthomas, hematological abnormalities (including macrothrombocytopenia, anemia, and stomatocytosis), and cardiovascular damage are frequently reported in Chinese patients [14, 15], findings consistent with the case we described. Notably, T-Chol and LDL-C concentrations of the patient’s parents were slightly above the normal range. After 1 month of statin treatment, their cholesterol levels decreased to within the normal range. This observation suggests that their mild hypercholesterolemia is not associated with the *ABCG5* gene mutations carried by the patient.

Data from the Exome Aggregation Consortium (ExAC) indicate that approximately 1 in 220 individuals harbor a loss-of-function (LOF) mutation in either the *ABCG5* or *ABCG8* gene [16]. Given this prevalence, it can be inferred that the actual incidence of sitosterolemia is likely higher than the currently reported global rates; the low recorded frequency of the disease is presumably attributed to underdiagnosis. In clinical practice, this suggests that patients presenting with similar clinical features (e.g., unexplained hypercholesterolemia, xanthomas, or hematological abnormalities) may potentially have sitosterolemia and thus require further targeted testing (e.g., phytosterol measurement or genetic analysis) to confirm the diagnosis.

The management of sitosterolemia typically encompasses three key approaches, as outlined below:​(1) Dietary intervention: Restricting the intake of both cholesterol and phytosterols is beneficial for reducing serum cholesterol levels and promoting the regression of xanthomas. Therefore, patients with sitosterolemia should be advised to strictly limit phytosterol consumption, with a focus on incorporating cereals, vegetables, and fruits low in phytosterols into their diet. Studies have demonstrated that dietary control alone can reduce serum phytosterol levels by 30–40% [17]. (2) Pharmacological therapy: This primarily includes cholesterol absorption inhibitors (e.g., ezetimibe) and bile acid sequestrants (e.g., cholestyramine). Ezetimibe exerts its effect by binding to the Niemann–Pick C1-Like 1 (NPC1L1) protein in the intestinal mucosa, thereby inhibiting the absorption of both dietary and biliary phytosterols and cholesterol [18]. Long-term follow-up studies have confirmed that daily administration of 10 mg ezetimibe is safe and effective; notably, this intervention can also partially reverse hematological abnormalities (e.g., macrothrombocytopenia and hemolysis) in patients with sitosterolemia [19]. The treatment response observed in the present case further supports that ezetimibe may be particularly effective for patients with secondary hypercholesterolemia caused by sitosterolemia. (3) Surgical intervention: For select patients, partial or complete ileal bypass surgery has been shown to significantly reduce serum phytosterol levels [20]. In the current case, after 2 months of ezetimibe monotherapy, the patient’s serum LDL-C and T-Chol concentrations returned to normal ranges. However, long-term follow-up is still necessary to monitor the progression of vascular plaques and maintain optimal disease control.

Sitosterolemia frequently leads to accumulation of atherogenic lipids in arterial walls, thereby increasing the risk of premature atherosclerotic cardiovascular disease (ASCVD) [21]. Pathogenetically, sitosterolemia impairs the cellular excretion of both xenosterols and cholesterol; additionally, if patients consume a diet high in cholesterol and animal products during the disease course, this dietary factor may further contribute to the observed elevations in serum cholesterol levels. Therefore, for patients with sitosterolemia, even if their blood lipid levels return to the normal range following pharmacotherapy, long-term clinical follow-up remains essential to optimize patient prognosis.

A notable limitation of our study is that we did not measure the patient’s plasma sterol concentrations prior to initiating treatment. It is important to note that, even when patients with sitosterolemia are on statin therapy, plasma phytosterols may still accumulate—an issue for which there is currently no effective intervention to prevent the formation or progression of vascular plaques.

## Conclusion

We report a case of sitosterolemia in an adult Chinese male harboring novel compound heterozygous mutations in the *ABCG5* gene. Following treatment with ezetimibe (combined with a low-plant-sterol and low-cholesterol diet), the patient’s serum cholesterol levels were effectively controlled. Therefore, for patients presenting with early-onset xanthomas and hypercholesterolemia that responds poorly to statin therapy, it is essential to perform plasma phytosterol (xenosterol) detection promptly to facilitate the differential diagnosis of sitosterolemia.

## Data Availability

The data that support the findings of this study are available from the corresponding author (Huandong Lin) upon reasonable request.

## Abbreviations

ABCG5: Adenosine triphosphate-binding cassette subfamily G member 5
ABCG8: Adenosine triphosphate-binding cassette subfamily G member 8
NPC1L1: Niemann–Pick Type C1-Like1
T-chol: Total cholesterol
LDL-C: Low-density lipoprotein cholesterol
STSL: Sitosterolemia
GC–MS: Gas chromatography-mass spectrometry
BMI: Body mass index
